# Advances in Functional Pet Food Research: Health-Driven Ingredients, Nutritional Targets and Evidence-Based Claims

**DOI:** 10.3390/ani16081222

**Published:** 2026-04-16

**Authors:** Sujira Vuthisopon, Pitiya Kamonpatana, Khwanchat Promhuad, Atcharawan Srisa, Phanwipa Wongphan, Anusorn Seubsai, Phatthranit Klinmalai, Nathdanai Harnkarnsujarit

**Affiliations:** 1 College of Innovation and Industrial Management, King Mongkut’s Institute of Technology Ladkrabang (KMITL), Bangkok 10520, Thailand; sujira.vu@kmitl.ac.th; 2 Department of Food Science and Technology, Faculty of Agro-Industry, Kasetsart University, Bangkok 10900, Thailand; fagipyk@ku.ac.th; 3 Department of Packaging and Materials Technology, Faculty of Agro-Industry, Kasetsart University, Bangkok 10900, Thailand; khwanchatpromhuad@gmail.com (K.P.); atcharawan.sri@ku.th (A.S.); phanwipa.w@ku.th (P.W.); 4 Department of Chemical Engineering, Faculty of Engineering, Kasetsart University, Bangkok 10900, Thailand; fengasn@ku.ac.th; 5 Faculty of Agro-Industry, Chiang Mai University, Samut Sakhon 74000, Thailand; 6 Center for Advanced Studies for Agriculture and Food (CASAF), Kasetsart University Institute for Advanced Studies (KUIAS), Kasetsart University, 50 Ngam Wong Wan Rd., Latyao, Chatuchak, Bangkok 10900, Thailand

**Keywords:** functional food, pet food, probiotic, prebiotics, companion animals, canine, feline

## Abstract

Functional pet food has grown rapidly, in line with the accelerated humanization of pets, growing attention to relations between diet and health, and mounting sustainability awareness. The article provides a critical overview of recent developments and new trends in functional pet food, combining data from published works, patents and market-driven innovative companies. The current trends depict a transition from single-nutrient fortification to integrated nutrition interventions through modulation of gastrointestinal health, immunity, metabolism, cognition and age-associated conditions. Special attention is dedicated to probiotics, prebiotics, postbiotics, polyphenols and novel protein sources, as well as innovations in processing and delivery technologies. The review highlights ongoing issues on the relevance of study design, available long-term safety information and our capacity to mechanistically underpin claims with respect to function. Because this review maps clusters of innovation and clusters of underdeveloped knowledge, it offers a roadmap for the translational pathway from scientific discovery to commercialization. The results highlight a call for harmonized methods, longer duration studies and integrative omics-based approaches in order to improve the evidence basis formulation and responsible marketing of future functional pet food products following credible, safe and sustainable strategies.

## Simple Summary

Functional pet food is rapidly expanding as pets are increasingly regarded as family members. However, challenges remain regarding the consistency of scientific evidence, long-term safety, and the credibility of health claims. This review analyzes data from research articles, patents, recent commercial innovations, and product claim trends to clarify development strategies, key ingredients, and measurable physiological outcomes in pets. The findings show a change from single-nutrient supplementation toward integrated formulations combining multiple active components to enhance beneficial effects. The emphasis is on measurable outcomes including gut health and microbiome changes, immunological and inflammatory responses, metabolic and weight control, kidney function support, and skin and coat quality improvements. Patents and product claims are becoming increasingly targeted, including formulations for gut health, fecal odor control, weight management, and hypoallergenic diets, especially for adult pet health. This article outlines principles for developing reliable and commercially viable pet food concepts that align with market trends while promoting pet wellness for owners and industry.

## 1. Introduction

Pet food has evolved from a primary focus on meeting basic nutritional requirements toward health-oriented formulations with more explicit functional roles. This shift is closely associated with the growing trend of pet humanization, whereby companion animals are increasingly regarded as family members and their diets are expected to support health, longevity, and quality of life in ways analogous to human nutrition [1,2]. Consequently, functional pet food has become a rapidly expanding research and commercial domain, integrating concepts from nutritional science, microbiology, food processing, and sustainability.

Functional pet food can be defined as complete diets or supplemental products formulated to deliver targeted physiological benefits beyond basic nutrition, such as improving gastrointestinal health, modulating immune responses, supporting cognitive function, regulating metabolism, or mitigating age-related disorders. Scientific advances in microbiome research and nutritional immunology have highlighted the role of bioactive ingredients in shaping health outcomes in companion animals [3,4,5]. In parallel, these developments have been accompanied by more rigorous evaluations of ingredient safety, bioavailability, and long-term tolerability [6,7].

Health-driven innovation has also become a major force shaping functional pet food development, including a growing research emphasis on transitioning from conventional animal-derived proteins toward alternative protein sources such as insects, plant proteins, and agri-food by-products [8,9,10]. At the same time, advances in processing technologies, including precision extrusion, encapsulation, and controlled-release systems, have improved the stability and delivery efficiency of functional compounds within complex pet food matrices [11]. These technological innovations have broadened the feasibility of incorporating sensitive bioactives while maintaining palatability and overall nutritional balance.

The literature base for this review was assembled from peer-reviewed publications indexed in Scopus and Web of Science, complemented by patent searches using WIPO PATENTSCOPE and Google Patents. Commercialization trends were assessed using Mintel’s Global New Products Database (GNPD). The search and screening covered publications and patents within the defined review window, and product launch data were analyzed for 2016–2025. Search terms included combinations of “functional pet food”, “dog”, “cat”, “companion animal nutrition”, “microbiome”, “immune”, “metabolic”, “urinary”, “skin and coat”, “novel protein”, and related terms. Studies were selected based on their relevance to functional ingredients, physiological or health-related outcomes, and application to dogs and cats. The collected evidence was then organized thematically by health domain, product function, and translational relevance.

## 2. Scientific Publications on Functional Pet Foods

### 2.1. A Key Research Direction in Functional Pet Food Development

The scientific literature on functional pet foods summarized reveals three broad and interrelated directions: (1) ingredient development within sustainability and circular economy frameworks, (2) enhancement of functional attributes associated with gastrointestinal health, metabolic regulation, and immunocompetence, and (3) post-processing quality assurance and safety validation of commercial formulations. The published studies are methodologically diverse, ranging from controlled in vivo feeding trials and digestibility assessments—predominantly conducted with canine extruded kibble formulations—to in vitro fermentation models and food science studies focused on physicochemical and stability-related properties [12,13,14,15]. Across this literature, there is substantial variation in bioactive selection, processing strategy, product format, and measured outcomes [16,17,18,19,20,21,22]. So, these studies highlight the expanding scope of functional pet food research, while also indicating the need for careful interpretation because the available evidence differs considerably in study design, endpoint selection, and translational strength.

A key direction in functional pet food research is the incorporation of alternative protein sources and by-products to reduce environmental impacts while maintaining—or enhancing—nutritional value. The evidence summarized in Table 1, Table 2, Table 3, Table 4, Table 5 and Table 6 indicates that marine by-products, such as squid meal and shrimp hydrolysate, can provide high-quality protein with elevated methionine content and improve apparent digestibility in dog diets, although overall palatability may remain lower than that of a basal formulation [13]. In a similar vein, the use of locally produced fish waste hydrolysate and fish oil has been shown to substitute for shrimp hydrolysate and salmon oil without compromising acceptance or digestibility. These substitutions were also associated with EPA/DHA-related benefits, reflected by a higher erythrocyte omega-3 index, alongside reductions in fecal ammonia-N and valerate following consumption of extruded kibble [23]. Follow-up work extending the evaluation to cardiovascular- and immune-related biomarkers further suggests that replacing shrimp hydrolysate/salmon oil with fish hydrolysate/fish oil may lower plasma triglycerides and angiotensin-converting enzyme activity, with a trend toward reduced total cholesterol, while leaving systemic cytokines/adipokines, clinical cardiac structure/function, and fecal IgA largely unchanged. Reported shifts in the gut microbiota, including increases in *Fusobacterium* and *Ileibacterium*, may indicate potential gastrointestinal benefits, although causal interpretation warrants caution [24]. Another prominent novel protein category is insect-based ingredients. Studies evaluating protein hydrolysates derived from yellow mealworm (*Tenebrio molitor*), house cricket (*Acheta domesticus*), and mulberry silkworm pupae (*Bombyx mori*) support the feasibility of insect hydrolysates as emerging protein sources for dog foods [25,26]. In particular, cricket protein hydrolysate at approximately 2% inclusion has been reported to enhance palatability, whereas higher levels may introduce bitterness and influence certain metabolic indicators (e.g., glucose and BUN), despite demonstrating antioxidant functionality during storage [27]. In parallel, comparative evidence within plant protein strategies highlights the need for careful formulation when increasing plant protein in carnivorous species. Using a carnivore model, the inclusion of 20% soybean meal reduced protein and energy digestibility; although colonic fermentation increased, it did not compensate for nutrient losses occurring in the upper gastrointestinal tract [28]. Overall, the literature supports the feasibility of using alternative proteins and by-products in pet food formulations, particularly in dogs. However, the strongest evidence currently relates to digestibility, palatability, and selected metabolic or gastrointestinal markers rather than long-term clinical health outcomes. In addition, responses appear to be ingredient-specific, and improved sustainability does not necessarily guarantee equivalent nutritional functionality without careful formulation.

### 2.2. Functional Evidence by Health Promotion

Functional pet foods are increasingly evaluated based on measurable, physiologically verifiable health outcomes rather than on ingredient type alone. The scientific literature further indicates that the efficacy of functional pet foods spans multiple health-related outcomes, arising from complex interactions among diet, the gastrointestinal tract, metabolic regulation, and broader physiological systems in companion animals. Based on the synthesis of evidence summarized in Table 1, Table 2, Table 3, Table 4, Table 5 and Table 6, functional claims in the scientific literature can be organized by health promotion as follows.

#### 2.2.1. Gut Health and Microbiota Modulation

Gastrointestinal health is among the most extensively investigated endpoints in functional pet food research (Table 1). Commonly applied indicators include stool quality (fecal score), short-chain fatty acid (SCFA) production, fecal ammonia nitrogen, and shifts in fecal microbial community structure. Evidence from both in vitro and in vivo studies suggests that functional ingredients derived from plants, agricultural by-products, algal extracts, and yeast-associated components can modulate intestinal fermentation patterns, thereby influencing luminal pH, the generation of beneficial metabolites, and the overall balance of the gastrointestinal microbiota. These changes are closely linked to stool characteristics and broader measures of intestinal function [17,20,22,25,29,30,31,32]. For example, an in vitro fermentation study using canine fecal inocula evaluated substrates such as beet pulp, pectin, and cellulose and demonstrated that inocula derived from yeast-fed dogs yielded higher SCFA production and a greater pH reduction, particularly when fermentable substrates (e.g., beet pulp and pectin) were provided. These conditions were also associated with an enrichment of SCFA-producing taxa and reductions in potentially undesirable groups, including *Fusobacterium* and *Streptococcus*. In an in vivo context, the incorporation of a lentil pasta by-product as a replacement for rice in dog diets increased SCFA concentrations and lowered fecal pH, while attenuating postprandial glucose and insulin responses. Notably, these metabolic and fermentation-related effects were achieved without deterioration of fecal score, and diet acceptance increased in the highest inclusion group (LP100) [32]. Complementary evidence from snack supplementation studies further supports the relevance of gut-focused functional interventions. Wang et al. [22] reported that supplementation with *Chenpi* (*Citrus reticulata* cv. *Chachiensis*) powder reduced fecal odor and decreased malodorous fecal compounds (3-methylindole, H_2_S, and NH_4_^+^–N). The intervention was also associated with enhanced serum antioxidant enzyme activities (SOD, CAT, and GSH–Px), reduced MDA and inflammatory markers (IL–8 and IFN–γ), and increased fecal secretory IgA. Microbiota profiling indicated a shift toward a higher relative abundance of Bacteroidota and lower Firmicutes, alongside the enrichment of predicted pathways related to metabolic and genetic information processing. Overall, current evidence supports the potential of gut-directed functional ingredients to influence intestinal fermentation, selected microbiota-related outcomes, and fecal characteristics. However, the translational significance of these changes is not always clear. Improvements in SCFA production or taxonomic abundance do not necessarily indicate clinically meaningful benefits unless they are accompanied by consistent improvements in stool quality, tolerance, or overall health status.

#### 2.2.2. Immune Support/Anti-Inflammatory and Antioxidant Outcomes

A substantial proportion of functional pet food research targets immune support and the modulation of systemic inflammation and oxidative stress. Evidence in this domain is commonly evaluated using biochemical and immunological indicators, including cytokine profiles, measures of antioxidant capacity, and biomarkers of oxidative damage. Collectively, the literature suggests that marine-derived components, protein hydrolysates, and plant-based bioactives may influence immune-related signaling and inflammatory status at the systemic level, although the magnitude and consistency of effects vary across formulations, study designs, and endpoints [14,16,17,22,24,27,30,33,34,35,36,37]. Clinical nutrition studies also indicate that antioxidant-oriented strategies may be relevant to disease-focused functional diets. For instance, a renal protective formulation incorporating omega-3 fish oil, alpha-lipoic acid, and antioxidant-rich fruit and vegetable sources administered for six months in dogs was associated with a reduction in serum symmetric dimethylarginine (SDMA; −14.3%) and an increase in total serum protein (+6.7%), without a concomitant change in body weight [38]. In addition, emerging evidence supports the potential of specific bioactives as antioxidant supplements. Lee et al. [14] reported that low-molecular-weight polysaccharides derived from *Tremella fuciformis* exhibit antioxidant functionality and stress resistance potential, linked to insulin/DAF–16 signaling and metabolic modulation. In that study, supplementation reduced malondialdehyde (MDA; −69.59%) and increased antioxidant enzyme activities, including total superoxide dismutase (T–SOD; 2.22-fold), glutathione peroxidase (GSH–Px; 1.28-fold), and catalase (CAT; 0.53-fold). Beyond bioactives intended to act in vivo, antioxidant systems are also used to protect product quality and limit oxidative deterioration in processed pet foods. For example, the incorporation of antioxidant additives—such as blends of ethoxyquin and tocopherols—during manufacture of extruded dry dog food has been shown to modulate oxidation, delay oxidative degradation, and improve product acceptance and shelf-life stability [12]. Overall, the evidence indicates that immune- and oxidative stress-related outcomes represent a major axis of functional claims in the peer-reviewed pet food literature. However, interpretation across studies should consider differences in baseline health status, diet matrices, dose and duration, and the selection of biomarkers, all of which can influence observed effects and their translational relevance. Despite promising findings, this evidence base remains methodologically diverse. Many studies rely on cytokine profiles, antioxidant enzyme activity, or metabolomic signatures rather than clinical endpoints, which limits direct comparison across studies and complicates the interpretation of practical benefits in routine pet feeding contexts.

#### 2.2.3. Weight Management and Metabolic Markers

Weight management and the improvement of metabolic homeostasis represent another major health endpoint for functional pet foods, particularly given the rising prevalence of obesity and metabolic dysregulation in companion animals. Studies in this area commonly assess postprandial glucose and insulin responses, indicators of energy utilization, and lipid-related metabolic biomarkers. Overall, the available evidence suggests that incorporating ingredients with appropriate digestibility and fermentability profiles can help modulate metabolic responses without compromising acceptance or stool quality [14,19,38,39,40,41]. Within the studies summarized in Table 1, functional strategies relevant to metabolic health frequently intersect with gastrointestinal mechanisms. For example, supplementation with a marine botanical/algae blend (macroalgae–microalga mixture) in dog diets has been evaluated for effects on palatability, nutrient digestibility, and fecal microbiota/metabolite profiles, supporting a potential role of marine polysaccharides and phytonutrients in shaping the gut ecosystem [19]. Beyond gut-centered outcomes, several formulations extend functional claims to systemic metabolic markers and organ-related indices. Evidence related to lipid metabolism is illustrated by work using fish hydrolysate and fish oil, which increased EPA/DHA exposure and was associated with favorable shifts in lipid-related biomarkers, including reduced plasma triglycerides and lower angiotensin-converting enzyme activity, without apparent adverse effects on clinical measures of cardiac structure or function [24]. With respect to glycemic control, the incorporation of lentil-derived by-products has been reported to support the development of lower-glycemic-response diets, a feature of potential relevance for dogs at risk of obesity or metabolic syndrome [15]. Additional evidence highlights the feasibility of plant-derived functional interventions for weight-related endpoints. Cho et al. [39] reported that supplementation of dry diets with barley sprouts contributed to weight management and obesity reduction in dogs, accompanied by lower circulating leptin and no detrimental changes in hematological parameters or serum biochemistry. Although overall microbial diversity was not markedly altered, the intervention was associated with a decrease in Firmicutes and an increase in Bacteroidetes, resulting in a reduced Firmicutes/Bacteroidetes ratio. Functional inference suggested the enrichment of pathways related to carbohydrate and amino acid metabolism as well as vitamin/cofactor metabolism, consistent with metabolic remodeling in response to dietary intervention. Overall, the evidence suggests that selected carbohydrate sources, fiber-rich ingredients, and bioactive-rich formulations may contribute to improved metabolic control or body-weight regulation, particularly in dogs. However, the available studies vary substantially in design, and the current evidence is still insufficient to generalize these effects across product types, species, or long-term feeding conditions. However, the current evidence in this area remains dominated by canine studies, and corresponding cat-specific data, particularly in relation to carbohydrate metabolism and amino acid-related nutritional regulation, remain limited. This limitation is particularly relevant for cats, whose obligate carnivorous physiology may influence carbohydrate handling and amino acid-related nutritional responses differently from dogs. In addition, taurine-related nutritional considerations remain more central in feline functional nutrition than in canine formulations.

#### 2.2.4. Renal/Urinary-Related Nutritional Strategies

A subset of functional pet food research focuses on supporting renal health and urinary tract function through targeted manipulation of dietary composition, particularly protein quality, mineral balance, and key chemical properties of the diet. Studies in this area typically evaluate clinical and biochemical indicators such as blood urea nitrogen (BUN), creatinine, mineral homeostasis, and urinary parameters relevant to renal workload and urinary tract status. Collectively, the available evidence supports the role of preventive, nutrition-based formulation strategies in reducing renal burden and supporting urinary system function, although the evidence base remains relatively limited [38,42]. Hall et al. [38] reported that functional bioactives incorporated into a renal protective food (RPF), including omega-3 fish oil, alpha-lipoic acid, antioxidant-rich fruit and vegetable components, and high-quality protein, can support renal function and nutritional status in older dogs. In a randomized block design involving 81 senior dogs, with 30 adult dogs included as a comparator group, animals were fed RPFs containing different levels of functional bioactive supplementation (FF1 and FF2) over a six-month period. Across all formulations, the glomerular filtration rate (GFR) increased by approximately 13.0–16.9% relative to baseline. The formulation with the highest level of bioactive enrichment also produced the most pronounced reduction in renal risk indicators, including a 14.3% decrease in symmetric dimethylarginine (SDMA), together with a 6.7% increase in total serum protein, without detectable changes in body weight or overall body composition. These findings suggest that combining a high-quality protein with anti-inflammatory and antioxidant bioactives may help preserve protein status and lean mass in senior dogs while supporting renal function. Overall, the available evidence supports the potential of preventive dietary strategies for renal and urinary health, particularly through optimization of protein quality, mineral balance, and antioxidant support. However, this evidence base remains relatively narrow and is still dominated by a limited number of canine studies. This area is especially relevant to cats, yet the available evidence summarized here remains more limited for feline-specific functional nutritional strategies than for dogs. Additional studies across species, particularly in feline-relevant urinary and renal contexts, are needed to confirm the broader applicability of these findings.

#### 2.2.5. Skin and Coat

Skin health and coat quality are functionally relevant endpoints in pet food research. Functional pet food studies in this domain commonly assess physical indicators of skin condition, measures of coat appearance and tensile integrity, and biomarkers related to essential fatty acid status. Overall, the evidence supports roles for polyunsaturated fatty acids, high-quality proteins, and antioxidant constituents in maintaining the structural integrity and physiological function of the skin–coat system. Guo et al. [30] evaluated methylsulfonylmethane (MSM) as a functional ingredient by feeding kittens extruded diets supplemented with MSM at 0.2% or 0.4% for 65 days. The authors reported that MSM can be incorporated to support skin- and coat-related outcomes without detectable adverse effects on growth performance, clinical safety biochemistry, or gastrointestinal homeostasis. Importantly, MSM did not disrupt energy balance or nutrient utilization in the kittens. With respect to coat attributes, MSM supplementation was associated with structural improvements in hair fiber quality, evidenced by a time-dependent reduction in cuticle scale thickness that remained lower than in the control group, consistent with smoother and glossier hair characteristics. Microstructural assessment by scanning electron microscopy (SEM), together with elemental and amino acid profiling of keratin, further supported a role for MSM in promoting hair fiber structure while maintaining stable nutritional and biochemical status in circulation. Although these findings are encouraging, the current evidence base for skin- and coat-related functional outcomes remains more limited than that for gut or metabolic health. In addition, many reported benefits are based on structural or appearance-related indicators rather than long-term dermatological or clinical outcomes.

**Table 1:** Gut health and microbiota modulation.

Functional Ingredients	Food Process	Dog/Cat	Product	Study Type	Factors of Investigation	Method of Investigation (Quality & Safety)	Health Promotion	Major Finding	**Ref.**
Squid meal; shrimp hydrolysate	Extruded basal diet	Dog	Extruded adult dog diet	in vivo	Inclusion level; palatability; digestibility; fecal traits; microbiota	Proximate analysis; palatability test; in vivo digestibility; fecal fermentation and microbiota assessment	Sustainable marine proteins; gut fermentation and microbiota support	Improved nutrient digestibility; shrimp hydrolysate showed antioxidant potential; both ingredients modulated selected fecal fermentation- and microbiota-related outcomes, although palatability remained lower than the basal diet	[13]
Red lentil pasta by-product	Extrusion	Dog	Extruded dry dog food (kibble)	in vivo	Rice replacement; digestibility; fecal traits; glycemic response	Extrusion performance; in vivo digestibility; fecal metabolites; postprandial glucose and insulin	Low glycemic potential; gut fermentation support	Increased SCFAs, lowered fecal pH, and reduced postprandial glucose and insulin at high inclusion, with acceptable fecal quality and palatability	[15]
Shrimp (*Litopenaeus vannamei*) hydrolysate	Extruded isoproteic diet	Dog	Extruded dry diet	in vivo	Diet type; immune markers; glucose; fecal microbiota	Hematology and biochemistry; cytokines; immune cell analysis; fecal microbiota assessment	Immunomodulation; gut microbiota support	Improved selected immune-related and oxidative stress markers, lowered glucose, and altered fecal microbiota	[16]
*Saccharomyces cerevisiae* fermentation product (SCFP)	Extrusion	Cat	Dry extruded adult cat diet (kibble).	in vivo	Intake level; palatability; stool quality; digestibility; microbiome	Feeding trial; digestibility; fecal metabolites; fecal metagenomics	Gut health and immune-associated support	Maintained digestibility and microbial diversity, with improved stool firmness at selected time points and better preference at lower intake level	[17]
Yeast β-glucan blend	Extrusion	Dog	Extruded adult dog food (kibble)	in vivo	Β-glucan carry-through; stool quality; digestibility; fecal metabolites	Extrusion stability test; feeding trial; digestibility and fecal analyses	Intestinal health/immune-support ingredient	Β-glucan remained stable after extrusion, but no clear intestinal benefits were observed at the tested levels	[18]
Algae blend (*Ulva rigida*, *Fucus vesiculosus*, *Chlorella vulgaris*)	Extrusion	Dog	Extruded kibble with supplement	in vivo	Inclusion level; palatability; digestibility; fecal traits; microbiota	Palatability test; total feces collection; fecal fermentation and microbiota analysis	Gut fermentation and fecal quality support	Improved selected digestibility measures and increased fecal SCFA, with minor microbiota changes; highest inclusion reduced palatability preference	[19]
Chenpi powder	Snack supplementation	Dog	Functional snack/treat	in vivo	Antioxidant status; inflammatory markers; fecal odor; fecal sIgA; microbiota	Feeding trial; serum antioxidant and inflammatory assays; fecal odor compounds; microbiota analysis	Gut health; mucosal immunity; fecal odor reduction	Reduced fecal odor compounds, improved selected antioxidant and inflammatory markers, increased fecal sIgA, and shifted microbiota composition	[22]
Fish protein hydrolysate + fish oil	Extrusion	Dog	Dry complete adult dog food	in vivo	Palatability; digestibility; fecal traits; omega-3 status; coat quality	Palatability test; crossover feeding trial; digestibility; fatty acid and fecal metabolite analysis	Sustainable circular economy ingredients; GI-related benefits	Maintained digestibility and palatability, improved omega-3 status, and reduced fecal ammonia-N and valerate	[23]
Fish protein hydrolysate + fish oil	Extruded kibble	Dog	Complete extruded dog food	in vivo	Cardiometabolic markers; fecal IgA; microbiota	Crossover feeding trial; blood biomarkers; echocardiography; fecal IgA; microbiota analysis	Cardiometabolic and gut microbiome support	Reduced triglycerides and ACE activity, with no adverse cardiac effects; microbiota shifts suggested possible gut-related benefits	[24]
Cricket protein hydrolysate	Milled/hot-air dried	Dog	Dry dog food pieces	in vivo + storage stability	Inclusion level; palatability; health markers; storage stability	Feeding trial; hematology and biochemistry; peroxide and acid value analysis	Alternative protein; antioxidant shelf-life support	Low inclusion improved intake, whereas higher inclusion reduced palatability; antioxidant protection during storage was dose-related	[27]
Antimicrobial peptides (AMPs)	Feeding trial	Cat	Complete cat diet	in vivo	Diarrhea; inflammatory markers; antioxidant status; microbiota; metabolome	Feeding trial; cytokine and antioxidant assays; fecal microbiota; serum metabolomics	Gut health; anti-inflammatory and antioxidant support	Reduced transport stress diarrhea and inflammatory markers, improved antioxidant status, and altered microbiota and metabolomic profiles	[31]
Whole egg powder; chondroitin sulfate	Hydrogel extrusion-based 3D printing	–	3D-printed functional pet snack (treat)	product development + in vivo model	Printability; ingredient dispersion; anti-inflammatory potential	Rheology; structure and texture analyses; OA mouse model	Functional delivery system; anti-inflammatory support	Improved printability and chondroitin sulfate dispersion; selected formulation showed anti-inflammatory effects in the OA model	[34]
Creatine and creatinine profile in commercial cat foods	Commercial extrusion	Cat	Dry extruded adult cat foods	commercial product analysis	Diet category; creatine/creatinine; amino acid adequacy	Nutrient composition and amino acid analyses	Nutrient adequacy/amino acid quality insight	Grain-free diets showed higher creatine and creatinine, while amino acid adequacy depended on formulation and reference pattern	[43]
Essential and non-essential elements	Commercial dry vs. canned products	Dog	Adult maintenance commercial dog foods	commercial product analysis	Diet format; mineral adequacy; non-essential element occurrence	Elemental analysis and multivariate comparison	Mineral balance and safety monitoring	Mineral profiles differed between dry and canned products, with variability in essential elements and presence of some non-essential elements	[44]
Different dietary protein sources	Extrusion	Dog	High-protein adult maintenance diets	in vivo	Protein source; digestibility; fecal traits; fecal metabolites; microbiota	Feeding trial; digestibility; fecal metabolite and microbiota analyses	Protein selection and gut fermentation outcomes	Protein source influenced digestibility, stool quality, proteolytic metabolites, and microbiota composition	[45]

**Table 2:** Immune support, anti-inflammatory and antioxidant outcomes.

Functional Ingredients	Food Process	Dog/Cat	Product	Study Type	Factors of Investigation	Method of Investigation (Quality & Safety)	Health Promotion	Major Finding	**Ref.**
Low-molecular-weight polysaccharides from *Tremella fuciformis* (TFLP)	Freeze-drying	–	Potential antioxidant functional ingredient	in vivo model	Dose level; oxidative stress response; antioxidant enzymes; gene expression; metabolomics	Structural characterization; stress model; biochemical assays; gene expression and metabolomics	Antioxidant and stress resistance potential	Reduced MDA, increased antioxidant enzyme activities, and supported stress resistance through pathways related to antioxidant defense and metabolism	[14]
Shrimp hydrolysate	Extruded isoproteic diet	Dog	Extruded dry diet	in vivo	Diet type; immune markers; glucose; fecal microbiota	Hematology and biochemistry; cytokines; immune cell analysis; fecal microbiota assessment	Immunomodulation; gut microbiota support	Improved selected immune-related and oxidative stress markers, lowered glucose, and altered fecal microbiota	[16]
Chenpi powder	Fed daily alongside maintenance diet	Dog	Functional snack/treat	in vivo	Antioxidant status; inflammatory markers; fecal odor; fecal sIgA; microbiota	Feeding trial; serum antioxidant and inflammatory assays; fecal odor compounds; microbiota analysis	Antioxidant; anti-inflammatory; gut and mucosal support	Improved selected antioxidant and inflammatory markers, reduced fecal odor compounds, increased fecal sIgA, and shifted microbiota composition	[22]
Fish protein hydrolysate + fish oil	Complete extruded kibble diets	Dog	Complete extruded dog food	in vivo	Inflammatory markers; adipokines; lipid profile; ACE activity; cardiac biomarkers; fecal IgA; microbiota	Crossover feeding trial; blood biomarkers; echocardiography; fecal IgA; microbiota analysis	Sustainable omega-3-rich ingredients; cardiometabolic and microbiome support	Reduced triglycerides and ACE activity, with no adverse cardiac effects; microbiota shifts suggested possible gut-related benefits	[24]
Microalgae (*Chlorella vulgaris*, *Nannochloropsis oceanica*, *Tetradesmus obliquus*)	Top dressing with extruded diet	Dog	Extruded adult dog food with microalgae supplement	in vivo	Inclusion level; palatability; digestibility; fecal metabolites; microbiota	Palatability and digestibility studies; fecal metabolite and microbiota analysis	Sustainable functional supplementation; gastrointestinal and microbiota support	Maintained intake and digestibility overall, with selected improvements in protein digestibility and microbiota-related outcomes depending on microalgae type	[29]
Methylsulfonylmethane (MSM)	Extrusion	Cat	Extruded dry diet for kittens	in vivo	Growth; serum biochemistry; antioxidant status; hair quality; fecal microbiota; metabolome	Feeding trial; serum and hair analyses; fecal microbiota; SCFA and metabolomics	Skin/coat support with safety confirmation	Improved hair quality at low inclusion without adverse effects on growth, serum biochemistry, microbiota, or metabolomic profile	[30]
Dried brewer’s yeast product	Extrusion	Dog	Dry dog diet; in vitro fermentation model	in vitro	Inoculum source; fiber substrate; fermentation pattern; microbiota	In vitro fermentation; pH, SCFA, BCFA, and microbiota analysis	Gut health via saccharolytic fermentation and microbiota modulation	Increased SCFA production and reduced pH with fermentable substrates, alongside shifts toward more favorable microbial groups	[32]
*Botanical blend*	Co-incubation with oxytetracycline	Dog	Functional/nutraceutical diet concept	in vitro	Anti-inflammatory activity; cytokine modulation; mitigation of oxytetracycline-induced effects	PBMC assay; cytokine staining; flow cytometry	Anti-inflammatory and immunomodulatory potential	Showed immunomodulatory effects and reduced pro-inflammatory responses under the tested conditions	[33]
Millet–chicken nutri-cereal mix	Malting and boiling	–	Dry fortified cereal mix/powdered mix	storage study	Storage condition; nutritional stability; phenolic retention; microbial quality	Proximate analysis; phenolic and mineral analysis; microbial counts	Antioxidant-related nutritional quality during storage	Refrigeration better preserved phenolics, nutritional quality, and microbiological stability during storage	[36]
Fish oil, alpha-lipoic acid, fruits and vegetables, high-quality proteins	Feeding trial	Dog	Renal protective food	in vivo	Bioactive level; GFR; protein status; renal biomarkers	Randomized feeding trial; GFR and blood biomarker assessment	Renal function support; antioxidant-oriented nutritional strategy	Increased GFR and improved selected renal risk markers, especially at higher bioactive enrichment	[38]
Modified eggshell powder	Drying, thermal treatment, baking	Dog	Calcium-fortified dog biscuits	product development	Microbial safety; calcium release; inclusion level; product quality	Microbiology; SEM/XRD; Ca release; proximate and texture analyses	Calcium supplementation with safety and product functionality	Improved calcium fortification while maintaining acceptable product quality and microbial safety	[46]
Casein phosphopeptide–selenium chelate (CPPSe)	Feeding trial	Dog	Functional snack/immune-support supplement	in vivo	Immune markers; transcriptome; metabolome; pathway responses	CBC and cytokine assays; RNA-seq; metabolomics	Immune-support functional ingredient	Modulated immune-related genes, metabolites, and pathways, supporting an immunoregulatory role	[47]

**Table 3:** Metabolic regulation and weight management.

Functional Ingredients	Food Process	Dog /Cat	Product	Study Type	Factors of Investigation	Method of Investigation (Quality & Safety)	Health Promotion	**Major Finding**	**Ref.**
Red lentil pasta by-product	Extrusion	Dog	Extruded dry dog food	in vivo	Rice replacement; digestibility; fecal traits; glycemic response	Extrusion performance; in vivo digestibility; fecal metabolites; postprandial glucose and insulin	Low glycemic potential; gut fermentation support	Increased SCFA, lowered fecal pH, and reduced postprandial glucose and insulin at high inclusion, with acceptable fecal quality and palatability	[15]
Fish protein hydrolysate + fish oil	Extrusion	Dog	Dry complete adult dog food	in vivo	Palatability; digestibility; fecal traits; omega-3 status; coat quality	Palatability test; crossover feeding trial; digestibility; fatty acid and fecal metabolite analysis	Sustainable circular economy ingredients; metabolic support	Maintained digestibility and palatability, improved omega-3 status, and reduced fecal ammonia-N and valerate	[23]
Fish protein hydrolysate + fish oil	Complete extruded kibble diets	Dog	Complete extruded dog food	in vivo	Lipid profile; ACE activity; inflammatory markers; microbiota	Crossover feeding trial; blood biomarkers; echocardiography; fecal IgA; microbiota analysis	Cardiometabolic support	Reduced triglycerides and ACE activity, with no adverse cardiac effects; microbiota shifts suggested additional gut-related benefits	[24]
Fish oil, alpha-lipoic acid, fruits and vegetables, high-quality proteins	Feeding trial	Dog	Renal protective food	in vivo	Bioactive level; GFR; protein status; renal biomarkers	Randomized feeding trial; GFR and blood biomarker assessment	Metabolic and renal support in aging	Increased GFR and improved selected renal risk markers, especially at higher bioactive enrichment	[38]
Barley sprouts	Dry diet supplementation	Dog	Anti-obesity/weight management dog diet	in vivo	Body weight; leptin; serum biochemistry; microbiota	Feeding trial; blood analyses; digestibility; fecal microbiota analysis	Weight management/anti-obesity	Reduced body weight and leptin without adverse hematological or biochemical effects; microbiota changes suggested metabolic remodeling	[39]
High amylose rice (Dodamssal)	Formulated diet	Dog	Dry dog diet for obesity prevention/weight management	in vivo	Body weight change; BCS; feed intake; serum biochemistry	Controlled feeding trial; weekly body condition monitoring; blood analyses	Weight management/anti-obesity	Reduced body weight gain and supported short-term weight loss, with lower metabolizable energy and no major safety concerns	[40]
Dietary protein level with sucrose-adjusted palatability	Purified diet formulation	Dog	Isoenergetic purified diets	in vivo	Protein %ME; palatability adjustment; intake regulation	Diet selection trial; intake measurement; plasma amino acid analysis	Macronutrient selection and metabolic intake regulation	Dogs regulated protein intake within a target range, while palatability influenced energy selection and feeding behavior	[41]
Different dietary protein sources	Extrusion	Dog	High-protein adult maintenance diets	in vivo	Protein source; digestibility; fecal traits; fecal metabolites; microbiota	Feeding trial; digestibility; fecal metabolite and microbiota analyses	Protein selection and metabolic/gut outcomes	Protein source influenced nutrient utilization, stool quality, proteolytic metabolites, and microbiota composition	[45]

Abbreviations: ACE, angiotensin-converting enzyme; BCS, body condition score; GFR, glomerular filtration rate; IgA, immunoglobulin A; ME, metabolizable energy; SCFA, short-chain fatty acid.

**Table 4:** Renal, mineral and safety-related nutritional strategies.

Functional Ingredients	Food Process	Dog/Cat	Product	Factors of Investigation	Method of Investigation (Quality & Safety)	Health Promotion	**Major Finding**	**Ref.**
Fish oil, alpha-lipoic acid, fruits and vegetables, high-quality proteins	Feeding trial	Dog	Renal protective food	Bioactive level; GFR; protein status; renal biomarkers	Randomized feeding trial; GFR and blood biomarker assessment	Renal function support in aging	Increased GFR and improved selected renal risk markers, especially at higher bioactive enrichment, without adverse effects on body weight	[38]
Dietary protein level with sucrose-adjusted palatability	Purified diet formulation	Dog	Isoenergetic purified diets differing in % metabolizable energy from protein	Protein %ME; palatability adjustment; intake regulation	Diet selection trial; intake measurement; plasma amino acid analysis	Macronutrient regulation relevant to nutritional management	Dogs regulated protein intake within a target range, while palatability influenced energy selection and feeding behavior	[41]
Fermented soybean-based ingredients with or without *Lactococcus lactis*	Autoclaving, fermentation, drying and milling	Dog & cat	Soybean-based protein ingredients for pet foods	Ingredient type; amino acid digestibility; protein quality; limiting amino acids	Digestibility assay; amino acid analysis; DIAAS-like evaluation	Sustainable plant protein development; amino acid adequacy guidance	Fermented soybean ingredients showed high amino acid digestibility, while limiting amino acids differed by species and life stage, indicating the need for careful formulation or supplementation	[48]
Dried whole black soldier fly larvae (DBSFL)	Feeding trial	Cat	Complete diets for gestation, lactation, and growth	Diet type; reproductive performance; growth; digestibility; clinical safety	Veterinary examination; feeding trial; hematology, biochemistry, urinalysis; digestibility assessment	Sustainable insect protein supporting feline growth and reproduction	Supported normal gestation and lactation in queens and improved nutrient and amino acid digestibility in kittens without major safety concerns	[49]

Abbreviations: DIAAS, Digestible Indispensable Amino Acid Score; GFR, glomerular filtration rate; ME, metabolizable energy.

**Table 5:** Skin, coat and structural functional outcomes.

Functional Ingredients	Food Process	Dog/Cat	Product	Factors of Investigation	Method of Investigation (Quality & Safety)	Health Promotion	Major Finding	**Ref.**
Fish protein hydrolysate + fish oil	Extrusion	Dog	Dry complete adult dog food	Palatability; digestibility; fecal traits; omega-3 status; coat quality	Palatability test; crossover feeding trial; digestibility; fatty acid and fecal metabolite analysis	Sustainable circular economy ingredients; skin- and coat-related nutritional support	Maintained digestibility and palatability, improved omega-3 status, and supported selected gastrointestinal benefits without affecting coat quality adversely	[23]
Methylsulfonylmethane (MSM)	Extrusion	Cat	Extruded dry diet for kittens	Growth; serum biochemistry; antioxidant status; hair quality; fecal microbiota; metabolome	Feeding trial; serum and hair analyses; fecal microbiota; SCFA and metabolomics	Skin and coat support with safety confirmation	Improved hair quality at low inclusion without adverse effects on growth, serum biochemistry, microbiota, or metabolomic profile	[30]

Abbreviations: SCFA, short-chain fatty acid.

#### 2.2.6. Other Emerging Endpoints

Beyond the major health outcomes discussed above, recent studies have begun to report additional functional endpoints, including stress resilience and broader indicators of wellbeing in companion animals. However, evidence in this area remains limited, and further work is needed to confirm the reproducibility of reported effects and to clarify the underlying physiological mechanisms [31]. In parallel, the scope of “functional” claims in the contemporary pet food literature also extends to product-centered attributes, most notably palatability and quality/stability metrics—which are highly relevant to consumer acceptance and real-world use [15,17,27,29,41,43,44]. This is particularly evident in extruded products, where maintaining hardness, bulk density, and expansion characteristics is essential to ensure appropriate texture and handling. For example, the use of corn-fermented protein (CFP) was reported to yield lower digestibility and increased fecal mass relative to corn gluten meal (CGM), yet still enabled the production of kibbles with broadly comparable structural properties and acceptable palatability; notably, cats exhibited a preference for CFP over CGM in that study [45]. Similarly, a sodium-reduced pâté supplemented with collagen hydrolysate and Salicornia perennans demonstrated improvements in product quality by reducing syneresis, increasing cohesiveness, and lowering oxidation-related indices (e.g., TBARS, carbonyl content, and acid value), while also enhancing color stability despite salt reduction [46]. In line with these findings, the inclusion of cricket protein hydrolysate was associated with reduced peroxide value under accelerated storage conditions, although formulation at higher inclusion levels may require careful balancing due to potential palatability constraints [27]. Taken together, these emerging endpoints highlight the expanding scope of functional pet food research beyond traditional physiological markers alone. Nevertheless, many of these applications remain at an early stage and require stronger validation to confirm their practical relevance in routine feeding contexts.

### 2.3. Processing and Delivery Effects

Beyond the identity of functional bioactives, scientific evidence indicates that manufacturing processes and delivery formats play critical roles in determining ingredient stability, bioaccessibility, and the expression of functional effects in pet foods. The studies summarized in Table 1 reflect a wide range of production technologies and product formats, including extruded dry foods, wet foods, snack-type kibbles, and specialized formulations such as renal protective diets. Each format can differentially shape the physical, chemical, and biological properties of the final product, thereby influencing both product performance and the consistency with which functional ingredients are delivered [17,20,43,50,51,52,53,54]. Extrusion—an enabling technology for most dry pet foods—is frequently combined with functional ingredient fortification, including protein hydrolysates, antioxidant systems, and skin-and-coat-oriented actives. Although extrusion involves high thermal and shear inputs, several studies indicate that certain functional ingredients can retain measurable efficacy post-processing, as reflected in outcomes such as coat quality, clinical safety, biochemistry, and animal acceptance. Importantly, these effects appear to depend on inclusion level and the intrinsic stability of the active compounds within the food matrix, underscoring the need to consider matrix–process interactions when interpreting functional outcomes [17,30,32,33,55]. In the context of therapeutic diets, including renal-support formulations, the formulation design and manufacturing conditions themselves can act as an effective “delivery system” for multiple bioactives without compromising acceptance or nutritional status. Controlling the qualitative and quantitative composition of protein, lipid fractions, and antioxidant components under appropriate processing conditions may promote stability and sustained functional activity toward target systems (e.g., renal function or lipid metabolism) over extended feeding periods [14,20,38,39]. In parallel, food technology research emphasizes the impact of processing on post-production product quality attributes—such as hardness, bulk density, internal structure, and oxidative stability during storage. These parameters are not only determinants of shelf-life and safety, but also influence palatability, feeding behavior, and the reproducibility of functional ingredient intake across meals. Therefore, the functional performance of pet foods cannot be inferred from bioactive selection alone. Instead, it should be evaluated in conjunction with manufacturing processes, product format, and delivery strategy. Integrating nutritional science with food processing and product engineering is therefore essential for developing functional pet foods that deliver consistent, scientifically verifiable health outcomes.

### 2.4. Safety, Limitations, and Research Gaps

Although the scientific evidence summarized in this review supports the potential of functional pet foods to promote health across multiple domains, considerations of safety, study limitations, and knowledge gaps remain central to both product development and the substantiation of functional claims at an industrial scale. Importantly, “functional” outcomes in the contemporary literature are not limited to host physiology; they also include interventions aimed at improving product safety and mitigating post-processing contamination risks. For example, coating kibbles with organic acid mixtures containing HMTBa after extrusion (i.e., post-kill step) has been reported to reduce *Salmonella* and Shiga toxin-producing *Escherichia coli* (STEC) by multiple log units within 12–24 h and to suppress *Aspergillus flavus* during extended storage. Notably, the WD–MAX formulation achieved high efficacy at a lower inclusion level, highlighting the potential of targeted post-processing strategies for microbiological risk control [56]. In cats, antimicrobial peptides (AMPs) have also been proposed as functional additives to mitigate diarrhea associated with transport stress, with reported effects including reduced cortisol and inflammatory cytokines and concurrent shifts in microbiota composition and metabolite profiles [31]. Across the studies compiled here, safety assessments are most commonly conducted over short-to-mid time frames, and many reports indicate no adverse effects on growth performance, routine blood biochemistry, or diet tolerability when functional actives are included at appropriate levels. Nevertheless, long-term safety data remain comparatively limited, particularly in senior animals and in pets with underlying chronic conditions, where vulnerability to nutritional perturbations may be greater. Methodological limitations are also recurrent. Many functional pet food studies involve relatively small sample sizes and modest intervention durations, and they differ substantially in study design (e.g., species, breed, age, baseline health status, and selected outcome measures), which constrains cross-study comparability and the strength of generalized conclusions. Moreover, a sizable proportion of studies emphasize biomarker- or metabolite-based endpoints rather than long-term clinical outcomes, thereby limiting direct translation to real-world populations and to clinically meaningful benefit statements. From a mechanistic standpoint, while the literature provides supportive evidence that functional ingredients can influence the gut ecosystem, metabolic regulation, immune signaling, and selected target organs, mechanistic resolution at the molecular and systems levels is still incomplete. In particular, the interactions among multiple actives within the same formulation, and the extent to which manufacturing conditions and delivery systems alter bioaccessibility and stability, are not consistently addressed. Species-specific differences further complicate interpretation: dogs and cats exhibit meaningful physiological and metabolic distinctions, yet comparative evidence across species remains sparse and unevenly distributed. Future research priorities should therefore include well-powered, longer-duration trials with clearly defined and clinically relevant endpoints, alongside harmonized outcome measures that enable robust comparison across studies. Integrating multi-omics approaches (e.g., metagenomics, metabolomics, and proteomics) with conventional clinical and nutritional assessments would strengthen mechanistic inference and improve causal interpretation. Finally, the development of standardized frameworks for safety and efficacy evaluation that align with regulatory expectations will be critical for supporting credible functional claims and facilitating translation into commercial practice. Species imbalance remains a notable limitation in the current literature, as functional pet food studies are still more heavily weighted toward dogs than cats, despite important physiological and nutritional differences between the two species. This is particularly important in the case of cats, given their obligate carnivorous metabolism and distinct nutritional priorities, including taurine dependence and urinary health-related formulation needs.

**Table 6:** Product quality, processing and stability.

Functional Ingredients	Food Process	Dog/Cat	Product	Factors of Investigation	Method of Investigation (Quality & Safety)	Health Promotion	Major Finding	Ref.
Rendered animal protein meals with different oxidation levels; antioxidant treatments	Extrusion	Dog	Dry extruded pet food (kibble)	Protein meal type; oxidation level; storage time; sensory quality; owner acceptance	Sensory evaluation; consumer acceptance test; microbiological screening	Quality/shelf-life support through oxidation control	Antioxidant treatment slowed oxidative deterioration and improved product acceptability during storage	[12]
Spray-dried animal plasma (SDAP) vs. wheat gluten (WG)	Emulsification, steaming and sterilization	Cat	Wet pet food (chunks in gravy)	Binder type; digestibility; fecal quality	Feeding trial; total feces collection; nutrient digestibility assessment	Digestive support proxy through improved nutrient utilization	SDAP improved apparent nutrient digestibility compared with WG in wet cat food	[20]
Yellow mealworm meal (*Tenebrio molitor*)	Extrusion	Dog	Dry extruded dog food	Inclusion level; nutrient profile; fatty acids; amino acids; texture	Proximate, fatty acid, amino acid, and texture analyses	Sustainable protein source with acceptable product quality	Increased unsaturated fatty acids and maintained feasible product properties up to high inclusion, although some amino acids may require supplementation	[26]
Cricket protein hydrolysate	Milled/hot-air dried	Dog	Dry dog food pieces	Inclusion level; palatability; health markers; storage stability	Feeding trial; hematology and biochemistry; peroxide and acid value analysis	Alternative protein; antioxidant shelf-life support	Low inclusion improved intake, whereas higher inclusion reduced palatability; antioxidant protection during storage was dose-related	[27]
Cereal fiber sources + sugar beet pulp vs. fruit-derived fiber sources	Extrusion	Dog	Adult dog complete diets	Fiber source; digestibility; fecal score; SCFA; microbiota; blood markers	Crossover feeding trial; digestibility; serum and fecal analyses; microbiota assessment	Ingredient selection for gut, lipid, and immune-related quality outcomes	Fiber source influenced digestibility, microbiota composition, fermentation profile, and selected metabolic markers without major deterioration in fecal consistency	[35]
Dietary phosphorus sources (organic vs. inorganic phosphates)	Feeding trial	Dog	Experimental complete maintenance diets	Phosphorus source; mineral homeostasis; postprandial and urinary responses	Feeding trial; blood, urine, and fecal mineral balance analyses	Phosphorus safety relevance in dog food formulation	Inorganic phosphates disrupted calcium–phosphorus homeostasis more strongly than organic phosphorus sources, raising safety concerns	[42]
Fish-derived proteins, meals, and hydrolysates	Rendering/enzymatic hydrolysis	Dog	Fish ingredient characterization and dog diets	Ingredient type; composition; digestibility; palatability	Composition analysis; protein quality assessment; palatability testing	High-quality marine protein ingredient selection	Fish hydrolysates and meals showed good protein quality and palatability potential, depending on raw material and processing	[57]
Carrot bagasse with different thickeners	Molding (gummies)	Dog	Sauce and gummies (treat prototypes)	Thickener type; concentration; heating; texture; microbiology; acceptance	Texture, physicochemical, microbiological, and acceptance analyses	Nutritional value and palatability in novel treat formats	Formulation and heating influenced texture and stability, while both prototypes showed good canine acceptance	[54]
Corn-fermented protein (CFP) vs. CGM and SBM	Extrusion	Dog & cat	Complete extruded pet diets/kibbles	Protein source; extrusion properties; digestibility; stool quality; palatability	Extrusion trials; digestibility testing; fecal scoring; palatability testing	Sustainable coproduct use with acceptable processing performance	CFP supported acceptable kibble production and species-specific palatability, although digestibility was lower than CGM in dogs	[52]
Collagen hydrolysate + *Salicornia perennans* extract	Vacuum mixing and retort sterilization	Dog	Canned meat pâté	Sodium reduction; syneresis; texture; oxidation; color stability	Proximate, texture, oxidation, and color analyses	Sodium reduction with improved product stability	Enabled sodium reduction while improving cohesiveness, oxidative stability, and color retention	[58]
Brewed chicken protein (BCP)	Precision fermentation, spray-drying and extrusion	Dog	Dry extruded adult dog food	Inclusion level; GI tolerance; digestibility; fecal metabolites; microbiota; palatability	Long-term feeding trial; digestibility; fecal and blood analyses; microbiota assessment	Safety/tolerance with potential GI benefits	BCP was well tolerated up to high inclusion, improved selected digestibility measures, and reduced proteolytic fecal metabolites	[53]
HMTBa-based organic acid mixtures	Post-extrusion coating	–	Coated dry kibbles	Inclusion level; time-dependent pathogen and mold reduction; residual effect	Microbial challenge study; enumeration and efficacy modeling	Post-processing microbial risk control	Coating with HMTBa-containing organic acid blends reduced bacterial and fungal contamination during storage	[56]
Crambe seed defatted meal and phytochemical extract	Pressurized liquid extraction	–	Processed flour and extract for formulation use	Solvent composition; extract yield; phenolics; antimicrobial activity; functional properties	Composition, antimicrobial, and functional property analyses	Potential antimicrobial ingredient and high-fiber/protein formulation material	Selected extraction conditions improved phenolic recovery and functional properties while reducing undesirable compounds	[59]
Pork by-products, chicken viscera, mechanically separated chicken, salts and pH modifiers	High-moisture model system	–	High-moisture model pet food system	pH; salts; phosphate; water retention; texture	Water retention and texture analyses	Processing-related quality optimization in high-moisture systems	pH had the strongest effect on water retention and texture, while salts had more limited influence	[60]
Atlantic salmon by-products; immobilized Alcalase	Enzymatic hydrolysis	–	Salmon oil ingredient for pet food/nutraceutical use	Hydrolysis conditions; oil yield; oxidation quality; enzyme reusability	Yield optimization; oil quality assessment; characterization of immobilized enzyme	Ingredient quality and low-oxidation oil recovery	Optimized hydrolysis gave good oil recovery with low oxidation, and the immobilized enzyme was reusable	[61]
Cannabidiol (CBD)	Soft capsule production	Dog	CBD supplement (capsule) with kibble	Long-term tolerance; clinical chemistry; liver markers; urinalysis; wellbeing	Randomized long-term feeding study; clinical and biochemical monitoring	Safety/tolerability of chronic dietary CBD exposure	Daily CBD was generally well tolerated over six months, with transient ALP elevation but no clear adverse clinical effects	[62]

Abbreviations: ALP, alkaline phosphatase; BCP, brewed chicken protein; CBD, cannabidiol; CFP, corn-fermented protein; CGM, corn gluten meal; HMTBa, 2-hydroxy-4-(methylthio) butanoic acid; SCFA, short-chain fatty acid; SBM, soybean meal; SDAP, spray-dried animal plasma; WG, wheat gluten.

## 3. Global Patents on Functional Pet Food Formulations and Technologies

The patent landscape analysis (Table 7 and Table 8) highlights innovations in functional pet food technologies, with a strong emphasis on ingredient selection and process optimization to address specific health needs in companion animals. Early patents primarily focused on gastrointestinal health and palatability enhancement through the use of prebiotics, hydrolyzed proteins, and flavor-improving approaches. More recent patenting activity has increasingly shifted toward precision nutrition, microbiome modulation, and life stage-specific formulations. A notable trend is the growing integration of alternative and sustainability-oriented ingredients, including insect proteins, microalgae, oilseed by-products, and marine plants [10,19,26,29,49]. These materials are often combined with fermentation, freeze-drying, or low-temperature processing to preserve bioactivity and improve bioavailability. In addition, inventions targeting probiotics and the microbiome indicate a transition toward strain-specific, evidence-based functional claims, supported by safety assessments and in vivo validation. At the same time, patents aimed at aging, metabolic disorders, dermatological conditions, and joint health underscore a broader movement from general nutrition toward condition-oriented dietary strategies. Collectively, these developments suggest that functional pet food innovation is converging across health functionality, sustainability, and processing technologies, reflecting a wider shift in companion animal nutrition that parallels advances in human functional and precision nutrition. Paul [63] developed Maillard reaction products derived from enzymatically hydrolyzed water-soluble proteins extracted from black soldier fly larvae. The process begins with isolating the soluble protein fraction, followed by controlled hydrolysis using one or more peptidase enzymes. This approach provides dual-phase functionality: enzymatic digestion enhances protein solubility and digestibility, whereas the Maillard reaction improves flavor attributes and alters the protein’s chemical characteristics without the need for synthetic additives. Overall, the method supports scalable and sustainable utilization of alternative protein resources. Huang et al. [64] proposed a functional pet food formulation designed to support feline urinary tract health, with a particular emphasis on relieving urinary retention. The product combines insect-derived protein extracted from black soldier fly larvae, a sustainable and high-quality protein source, with conventional animal proteins, taurine, prebiotics, and essential micronutrients to enhance functional performance. The formulation further incorporates selected traditional Chinese herbal extracts that are freeze-dried to preserve heat-sensitive nutrients and bioactives, while also improving product stability and palatability. Kim et al. [65] developed a probiotic-based functional pet food ingredient intended to alleviate atopic dermatitis and other inflammation-associated skin disorders in companion animals. In vivo testing using an atopic dermatitis model further showed marked reductions in epidermal thickness and inflammatory lesions, together with observable improvements in overall skin condition after oral administration. Jeong et al. [66] described a cooked functional pet food formulation incorporating desalinated seaweed extracts, then vacuum packaged and sterilized via retort processing. Seaweed-derived materials increase antioxidant capacity and dietary fiber content. The patent does not clearly define the responsible bioactive compounds or outline a standardization approach, which may limit reproducibility and consistent functional performance across raw material batches. Lin et al. [67] reported a functional pet food formulation incorporating carotenoids derived from a radiation-resistant *Deinococcus* strain under low-temperature conditions. The patent claims that these carotenoids enhance systemic antioxidant capacity, mitigate age-associated oxidative stress, and help support a balanced gut microbiota in cats. Xia et al. [68] developed a functional pet food by blending animal-derived protein, crude plant protein, and insect-based protein sources, formulated in combination with dietary fiber and probiotic components. The production process includes fermentation followed by twin-screw extrusion, aiming to enhance protein digestibility, improve the stability of lipid dispersion, and optimize texture and palatability. The product is subsequently surface-coated and freeze-dried. Liu et al. [69] developed a probiotic-based functional ingredient for dog food using a *Pediococcus acidilactici* strain isolated from the gastrointestinal tract of healthy dogs. The strain was cultivated under controlled anaerobic conditions and preserved via freeze-drying. Comprehensive safety evaluation further indicated the absence of virulence-associated traits and transferable antibiotic-resistance genes, positioning the ingredient as a potential alternative to conventional antibiotic-based approaches for maintaining canine gut health. Moon [70] introduced a functional pet treat formulated to support joint health in companion animals. The core recipe uses white-fleshed fish as the primary protein source. The formulation may also include botanical extracts and supplemental nutrients. These components are intended to modulate inflammatory responses, support metabolic function, and enhance palatability. Sang-gyu et al. [71] developed fish oil, protein hydrolysates, and bone-derived materials, from aquatic processing by-products. The process employs hydrolysis followed by decantation-based fractionation to separate the oil fraction and the protein hydrolysate. Lee [72] described a liquid functional supplement for pets formulated from green-lipped mussel, shark cartilage, marigold, barley sprouts, and gardenia using low-temperature pasteurization. The product was developed in a stick-type sachet format to enhance palatability and voluntary intake that supported gastrointestinal function, joint integrity, skin and eye health, and cognitive performance. Hong and Jo [73] incorporated fermented black soldier fly (*Hermetia illucens*) larval extracts into a functional pet food formulation. The patent claims that the resulting extract supports gastrointestinal health, helps modulate immune responses, and improves nutrient absorption. Chen [74] described the development of a functional pet food that combines probiotics with plant-derived digestive enhancers, with controlled extraction and drying processes used to prepare the herbal components. The formulation is intended to promote gastrointestinal balance and improve nutrient absorption while avoiding synthetic additives or antibiotic use. Overall, the invention reflects a broader shift toward clean-label approaches in pet food development. Liu et al. [75] represented a functional pet food that uses freeze-dried meat as the primary delivery matrix for a blend of antioxidants, probiotics, prebiotics, digestive enzymes, and metabolism-regulating ingredients. The manufacturing process integrates a two-step freeze-drying strategy with cyclodextrin inclusion technology to encapsulate sensitive components while maintaining sensory acceptance. Cambou and Niceron [76] proposed the use of free sulfur-containing amino acids as functional components to enhance palatability in pet food formulations without fat. The invention focuses on incorporating cysteine analogs and methionine derivatives for dry and wet products. Lee [77] described a functional snack designed to mitigate taurine deficiency in cats by incorporating an elevated taurine level together with animal-derived proteins, using freeze-drying and low-temperature processing. Park [78] proposed a model for producing personalized functional pet food based on individual health data obtained from genetic analysis and veterinary assessment. The process involves applying a customized surface-coating formulation (palatability enhancers from animal- and plant-derived oils) after shaping and drying. Ahn et al. [79] formulated a functional pet food using fresh venison as the primary protein source, supplemented with a broad set of animal- and plant-derived ingredients. The formulation is intended to support overall metabolism and essential nutrient intake, promote digestion, improve palatability, and contribute to skin health. Yoon and Park [80] proposed the use of oilseed cakes, *Amaranthus mangostanus* and *Indigofera pseudotinctoria.* They are designed to enhance satiety while supporting metabolic and immune health. Huang et al. [81] developed cat feed that accommodates nutritional requirements varying by breed, age, body size, and specific health status. Animal- and marine-derived protein components are combined in adjustable ratios (modular pellet concept), which support gastrointestinal health, immune function, urinary tract function, and hairball control, while reducing reliance on synthetic additives. Lee et al. [82] utilized fucoxanthin-rich microalgae with animal-derived proteins and fats for obesity and diabetes in pets. The process combines fluidized-bed drying with spray-coating and then produces pellet- or tablet-type products. Kim and Hong [83] developed a pet treat for oral health by reducing the accumulation of dental plaque and tartar. The product uses a meat base with plant extracts from *Agastache rugosa*, *Portulaca oleracea*, and stevia (antimicrobial and antioxidant properties). Processing involves blanching followed by low-temperature drying. Ratuld [84] investigated a process for producing palatability-enhancing protein hydrolysates for pet food. The process used enzymatic hydrolysis of animal-derived muscle, skin, bone, and other processing residues, using alkaline endopeptidases under controlled pH conditions. Flavor development uses the Maillard reaction. The invention of Yoshino et al. [85] focuses on processing approaches that preserve the physiological activity of functional components while ensuring stability and compatibility within conventional food matrices. Particular emphasis is placed on maintaining sensory quality and overall product integrity throughout formulation and production. Wu and Yang [86] developed a method for producing functional dog biscuits incorporating fructooligosaccharides (FOSs) as a prebiotic. The formulation uses a defined flour blend of wheat flour, corn flour, and sweet potato flour at a 3:2:1 ratio, with FOS added at 8–10% (*w*/*w*). The patent indicates that FOS exerts its functional effect by selectively promoting beneficial gut microbiota, thereby supporting digestive performance, nutrient absorption, and immune health. Hodge et al. [87] applied *Yucca schidigera* extract as a natural deodorizing agent in animal feed formulations, with the primary aim of reducing fecal odor and ammonia emissions. The products are described in solid and semi-solid forms. Beyond odor control, the patent also suggests potential benefits in modulating the intestinal environment, which may contribute to overall digestive health.

From the patent data, it was found that there is no clear comparative comparison on fundamental dietary requirements between cats and dogs. Most formulas made especially for cats include taurine supplementation to prevent taurine deficiency and related disorders such as retinal degeneration and cardiomyopathy in cats [77]. Additionally, formulas combine taurine with other ingredients to support urinary tract health in cats [64]. While the patent for dogs does not address taurine deficiency as a key issue due to the differing needs of dogs and cats, it focuses on other functional targets, i.e., breed-specific probiotics for gut microbiome balance [69] and the use of FOSs in dog biscuits to promote gastrointestinal health [86].

## 4. Mintel GNPD Market Overview

The Mintel Global New Products Database (GNPD) is a commercial database used to track food product launches and market trends, including the use and positioning of additives and ingredients. In this section, Mintel GNPD data were used to analyze recent market trends in functional pet food launches based on on-pack claims, with particular attention to how claims, nutrient content, and ingredient positioning vary across complete diets, snacks and treats.

### 4.1. Functional Health Claims

**Figure 1:**
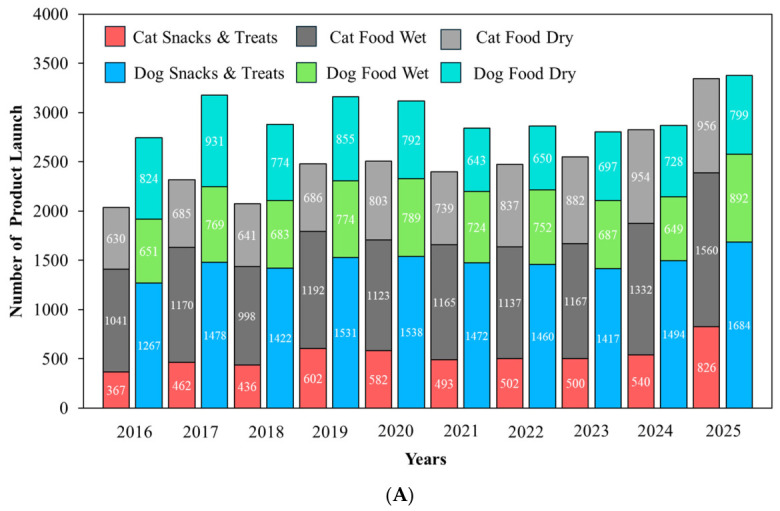

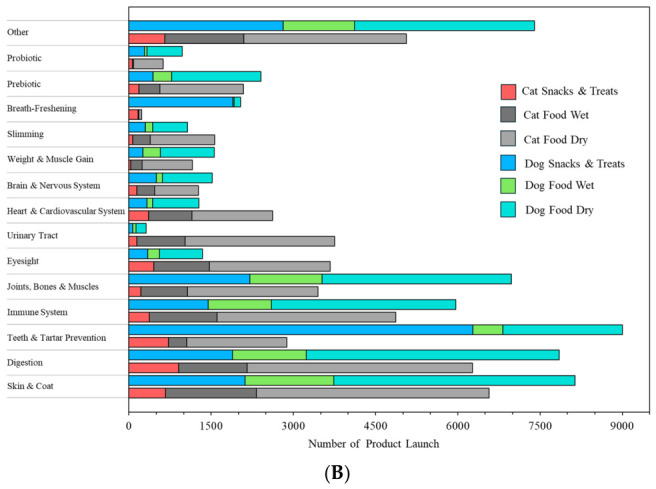
Numbers of functional pet food launches recorded in Mintel GNPD between January 2016 and December 2025 in the following “Pet Food” sub-categories: “Cat Food Dry”, “Cat Food Wet”, “Cat Snacks & Treats”, “Dog Food Dry”, “Dog Food Wet”, or “Dog Snacks & Treats”. (**A**) Launches carrying one or more of the claims “Functional” or “Functional Pet”. (**B**) Launches carrying one or more Functional Pet claim categories, including Immune System; Teeth & Tartar Prevention; Joints, Bones & Muscles; Anti-Parasite; Weight & Muscle Gain; Digestion; Brain & Nervous System; Eyesight; Skin & Coat; Slimming; Heart & Cardiovascular System; Urinary Tract.

Figure 1A shows that Mintel GNPD pet food launches carrying a “functional” or “functional pet” increased steadily between January 2016 and December 2025 (*n* = 54,834). Annual launches increased from 4780 in 2016 to 6717 in 2025 (+40.5%), while cat snacks and treats showed the strongest growth, rising from 367 to 826 launches (+125.1%). Cat food wet accounted for 11,885 launches and increased to 1560 in 2025 (+49.9%) and cat food dry also expanded (+51.7%), consistent with up-formulation of dry diets for health positioning. Dog food wet grew by 37.0%, whereas dog food dry declined slightly (−3.0%), suggesting relative saturation of standard dry formats. Dog snacks and treats contributed the largest volume (14,763 launches) and reached 1684 launches in 2025 (+32.9%), supporting its role as a fast-cycle format for line extension and claim renewal. Overall, the highest innovation and clearest claim signaling concentrates on snacks, treats and wet products.

Figure 1B shows the distribution of the 15 most frequent functional topics across six product sub-categories. Skin and coat was the most common claim with 14,715 launches, followed by digestion with 14,122 launches. Both themes were driven mainly by dry diets. For skin and coat, dog food dry contributed 4404 launches and cat food dry contributed 4243. For digestion, dog food dry contributed 4617 launches and cat food dry contributed 4113. These patterns indicate that core diets are the main vehicle for system-level nutrition positioning.

By contrast, some claims were more clearly associated with snacks and treats or species-specific formats. Teeth and tartar prevention reached 11,883 launches and concentrated on dog snacks and treats (6273), while breath freshening also peaked in dog treats (1896), supporting treats as a rapid format for faster cycling of on-pack claims and texture-enabled mechanical functions. Urinary tract claims totaled 4069 and clustered in cat food dry (2729), consistent with the demand for condition-specific feline formulations. Immune system (10,840) and joints, bones and muscles (10,431) also remained substantial, whereas prebiotic (4497) and probiotic (1602) claims were smaller, highlighting the need for stronger clinical evidence and improved delivery technologies to support claim credibility.

### 4.2. Functional Ingredient Claims

**Figure 2:**
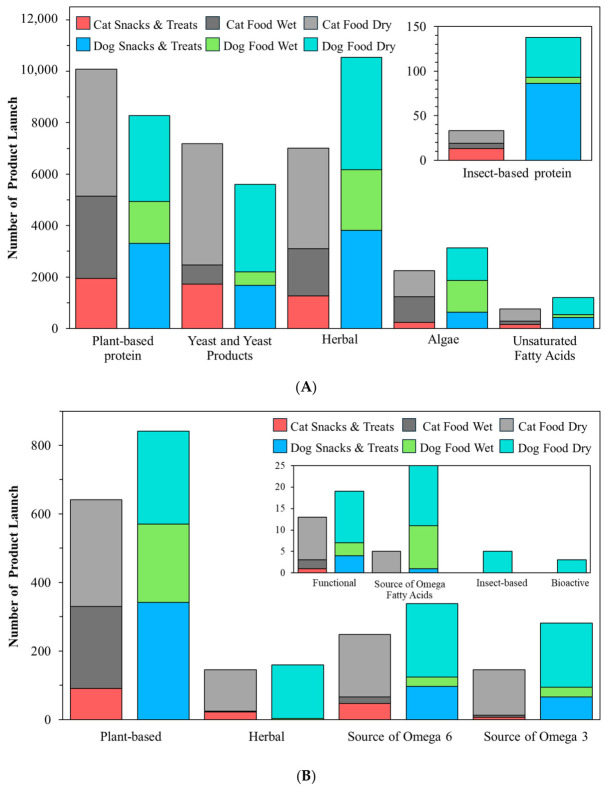
Numbers of functional pet food launches recorded in Mintel GNPD between January 2016 and December 2025 in the following “Pet Food” sub-category: “Cat Food Dry”, “Cat Food Wet”, “Cat Snacks & Treats”, “Dog Food Dry”, “Dog Food Wet”, or “Dog Snacks & Treats”. All launches carried one or more of the claims “Functional” or “Functional Pet”. (**A**) Ingredient search matches one or more of the following: Plant Protein; Yeast and Yeast Products; Herbal Preparations; Herbal Substances; Algae; Unsaturated Fatty Acids; Insect Protein. and (**B**) Ingredient claims search matches one or more of the following: Plant Based; Insect Based; Functional; Herbal; Plant Based; Source of Omega 3; Source of Omega 6; Source of Omega Fatty Acids; Rich in Unsaturated Fatty Acids; Bioactive.

Figure 2A summarizes functional ingredient use across Mintel GNPD functional pet food launches from 2016 to 2025, whereas Figure 2B shows which of these ingredients were explicitly promoted as ingredient claims. In Figure 2A, plant-based protein showed the widest adoption and was concentrated in cat food dry (4948), dog food dry (3338), and dog snacks and treats (3304). Typical claim wording highlights blends of plant- and animal-derived ingredients, vegan formulations, and high-quality plant protein sourcing. A recent synthesis of publications and Mintel GNPD trends reports that soy, wheat, and pea proteins dominate plant protein use in pet foods, and pea protein is increasingly adopted as a lower-allergen alternative in grain-free or soy-free recipes [10]. This aligns with Figure 2A, where plant-based protein shows broad uptake and is often positioned through blended plant–animal formulas or vegan sourcing cues.

Yeast and yeast products also scaled strongly in dry formats, led by cat food dry (4722) and dog food dry (3410), and were commonly described as sources of protein, B vitamins, and minerals that support coat condition, immune support, and palatability. Herbal ingredients are widely used across core diets and treats, led by dog food dry (4365), dog snacks and treats (3816), and cat food dry (3902), and were typically framed as botanicals for natural and holistic wellbeing. Algae appeared at a moderate volume and is led by dog food dry (1269) and dog food wet (1237), with descriptions highlighting spirulina and marine-derived EPA and DHA to support gut health and broader wellbeing. Unsaturated fatty acids are smaller in scale and are led by dog food dry (659) and cat food dry (465) and were mainly positioned as omega-rich support for coat shine and skin health, sometimes paired with DHA and EPA or linked to prebiotic and probiotic concepts. Insect protein remains niche and is concentrated in dog snacks and treats (86), with messaging that emphasizes digestibility and hypoallergenic suitability for sensitive dogs. A recent GNPD-focused review (2014–2024) similarly notes that insect proteins are used mainly in dog products, especially snacks and treats, and are marketed as hypoallergenic and highly digestible [10].

Figure 2B shows that only some commonly used functional ingredients were consistently elevated to ingredient claim cues. The plant-based claim is dominant (1482) and spanned core diets and snacks, led by cat food dry (311), dog snacks and treats (342), dog food dry (271), dog food wet (228), and cat food wet (239), indicating that plant-based positioning is used for both daily feeding and treat-led trials. A source of omega-6 was the second-largest claim (586) and was concentrated in dry foods with meaningful treat uptake, led by dog food dry (214), cat food dry (182), and dog snacks and treats (97), consistent with a simple fatty acid cue widely used for skin and coat messaging. The Source of omega-3 claim is also common (426) and shows a similar distribution, led by dog food dry (187) and cat food dry (132), with smaller volumes in wet foods and snacks. Botanical and herbal claims are moderate in scale (306) and concentrate on dog food dry (156) and cat food dry (121), while treats contribute little overall (dog snacks and treats = 0; cat snacks and treats = 22), suggesting that botanical messaging is more often embedded in staple diets than used as a primary snack claim. In contrast, direct ingredient claims such as “functional” (32), “source of omega fatty acids” (30), “insect-based” (5), and “bioactive” (3) are limited, and algae- or yeast-specific claim cues are rare. These findings suggest that several ingredients are widely used in formulations, but only a smaller number are consistently translated into front-of-pack ingredient claims.

### 4.3. Functional Nutrition Claims

**Figure 3:**
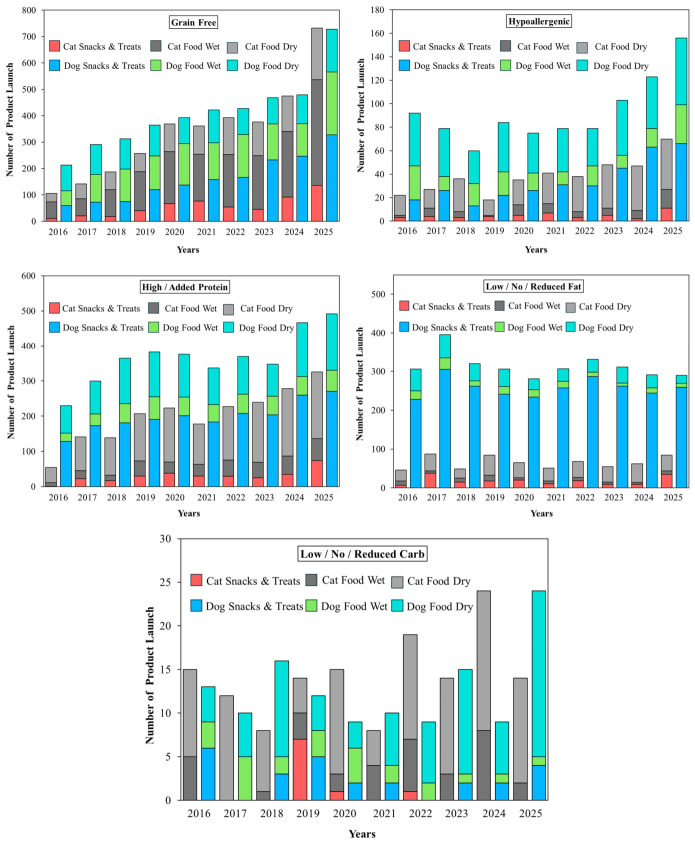
Numbers of functional pet food launches recorded in Mintel GNPD between January 2016 and December 2025 in the following “Pet Food” sub-category: “Cat Food Dry”, “Cat Food Wet”, “Cat Snacks & Treats”, “Dog Food Dry”, “Dog Food Wet”, or “Dog Snacks & Treats”. Launches carried one or more of the following claims: “Functional”; “Functional Pet”; “grain-free”; “hypoallergenic”; “high/added protein”; “low/no/reduced fat”; “low/no/reduced carb”.

Figure 3 quantifies nutrition positioning claims within functional pet food launches in the Mintel GNPD from 2016 to 2025 and shows that grain-free and protein-forward claims exhibited the clearest expansion. Grain-free showed the fastest growth, led by cat food wet (62 to 400; +545.16%) and cat food dry (33 to 196; +493.94%), with strong increases in dog snacks and treats (59 to 328; +455.93%) and dog food wet (57 to 238; +317.54%), and cat snacks and treats rising from 11 to 136 (+1136.4%), whereas dog food dry increased more slowly (97 to 162; +67.01%), consistent with a more mature dry segment. Grain-free launches are often framed for pets with grain-related sensitivity or digestive tolerance. Hypoallergenic properties also grew in dog snacks and treats (18 to 66; +266.67%) and cat food dry (17 to 43; +152.94%); cat food wet increased from 2 to 16 (+700.00%) but remains small in absolute volume, and cat snacks and treats increased from 3 to 11 (+266.67%). This messaging commonly emphasizes the exclusion of common allergens and is frequently co-declared with “grain-free”, “no grain”, or “single-protein/monoprotein”. High/added protein also showed acceleration, especially in cat products, with cat food wet increasing from 10 to 62 (+520.00%) and cat food dry from 43 to 189 (+339.53%), while dog snacks and treats remained a major outlet (128 to 270; +110.94%) that supports protein-forward differentiation in fast-cycle formats. Cat snacks and treats expands from 1 to 74 (+7300.0%) from a low baseline.

By contrast, low/no/reduced fat was the highest-volume nutrition claim in dog snacks and treats (2579 launches), but its growth was modest, increasing from 228 in 2016 to 259 in 2025 (+13.60%). Dog food dry declined from 56 to 21 (−62.50%) and dog food wet declined from 22 to 10 (−54.55%), whereas cat snacks and treats increased from 7 to 35 (+400.0%) but remained small in absolute volume. Low-fat positioning is often paired with high-protein formulation to support the maintenance of muscle mass and proper body weight. Low/no/reduced carbs remain niche overall. Dog food dry increased from 4 to 19 (+375.00%), whereas wet and snack formats generally declined, and cat snacks and treats contributed only nine launches across the full period. This pattern indicates an early stage, dry-led positioning that may require clearer clinical rationale and feeding guidance to scale.

### 4.4. Functional Quality/Safety Claims

**Figure 4:**
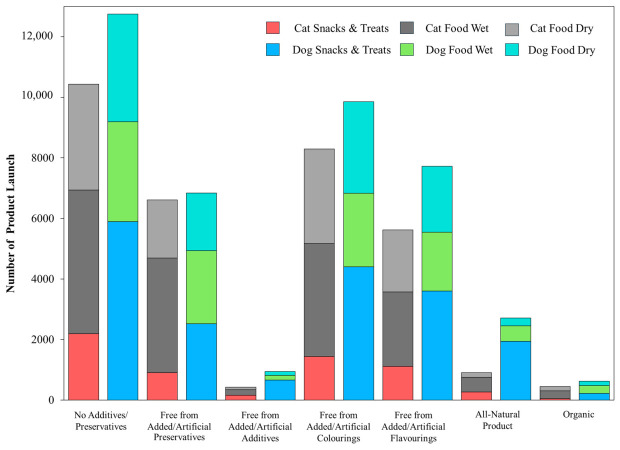
Numbers of functional pet food launches recorded in Mintel GNPD between January 2016 and December 2025 in the following “Pet Food” sub-category: “Cat Food Dry”, “Cat Food Wet”, “Cat Snacks & Treats”, “Dog Food Dry”, “Dog Food Wet”, or “Dog Snacks & Treats”. Launches carried one or more of the following claims: “Functional”; “Functional Pet”; No Additives/Preservatives; Free from Added/Artificial Preservatives; Free from Added/Artificial Additives; Free from Added/Artificial Colorings; Free from Added/Artificial Flavorings; All-Natural Product; Organic.

Figure 4 summarizes quality and safety claims within functional pet food launches in the Mintel GNPD from 2016 to 2025 and shows that clean-label cues were the dominant positioning strategy. “No additives/preservatives” was the leading claim (23,180 launches), led by dog snacks and treats (5896) and cat food wet (4745). By 2025, these formats reached 709 and 663 launches, respectively, while cat snacks and treats also scaled to 357 launches in 2025. “Free from added/artificial colorings” ranked second (18,148) and expanded across formats, notably in cat food wet (255 to 521) and dog snacks and treats (300 to 461), with cat snacks and treats reaching 230 launches in 2025. “Free from added/artificial preservatives” (13,450) and “free from added/artificial flavorings” (13,344) showed a similar scale and further reinforce a clean-label narrative, particularly in wet foods and treats.

Illustrative on-pack wording included claims such as “free from preservatives and colourants” for complete adult dog food, “free from added colourings and food attractants, light on the stomach” for complete cat food, and “free from additives, added preservatives, and food attractants” for natural pet treats. “All-natural product” remained moderate (3613) but rises in dog snacks and treats (129 to 193), whereas “free from added/artificial additives” (1366) and “organic” (1070) remained niche. These patterns suggest that most launches prioritize simple avoidance language over certification. A systematic review found no harm from approved additives or preservatives within regulatory limits, indicating that clean-label messaging should avoid over-claiming and be evidence-based [88].

Across the Mintel GNPD analysis (2016–2025), differences between dogs and cats were reflected more clearly in claim positioning than in the direct identification of basic nutritional requirements. Urinary tract claims were concentrated in cat food dry (2729), consistent with the development of formulas targeting urinary health in cats. In contrast, teeth and tartar prevention and breath-freshening claims were more concentrated in dog snacks and treats, reflecting the use of snack formats in dogs for oral health purposes. By comparison, ingredient claims such as plant-based protein, omega-3 or 6, and yeast were used in both dogs and cats without clear species-specific nutritional separation. Therefore, this section highlights how functional claims are positioned according to the health priorities more commonly emphasized in dogs versus cats.

## 5. Conclusions

The rapid maturation of functional pet food research reflects a shift from ingredient-centered concepts toward integrated, evidence-based nutritional strategies that combine sustainability, physiological relevance, and product performance. Across scientific publications, patents, and market data, functional innovation is increasingly characterized by the convergence of alternative protein sources, gut microbiota modulation, immune and metabolic support, and condition-specific nutrition, supported by advances in processing and delivery technologies. Importantly, the evidence underscores that functional efficacy cannot be inferred from ingredient selection alone; rather, it emerges from the interactions among formulation design, processing conditions, bioactive stability, and delivery format. While short- to mid-term studies generally support safety and potential health benefits, limitations remain regarding long-term outcomes, mechanistic understanding, and cross-species comparability. Future progress will depend on well-powered, longer-duration trials with harmonized endpoints, alongside multi-omics approaches that strengthen causal interpretation and translational relevance. Aligning scientific validation with regulatory frameworks and market expectations will be essential to ensure that functional claims remain credible, meaningful, and sustainable as the sector continues to evolve.

## Figures and Tables

**Table 7:** Invention patents on functional pet food formulations and technologies from protein sources.

Functional Ingredients	Food Process	Product or Target Application/Major Claim	Country of Patent Applicants	**Patent Name**	**Ref.**
Soluble black soldier fly larvae protein	Enzymatically hydrolyzed	Water-soluble insect proteins: Maillard reaction products of enzymatically hydrolyzed water-soluble black soldier fly larvae protein obtained	USA	Hydrolysate of water-soluble insect proteins and method for preparation thereof	[63]
Black soldier fly larvae protein, natural steroid saponin, rosemary extract	Freeze-drying	Functional pet food targeting cat urinary retention: Combines high-quality insect protein with Chinese herbal extracts and micronutrients to assist in treating adult cat urinary retention, improve immunity, promote beneficial gut flora, enhance palatability, and offer a low-cost, nutrient-dense functional diet	China	A functional pet food for cat urinary retention based on insect protein converted from organic waste and compounded with Chinese herbal medicine	[64]
Animal meat paste, plant crude protein, insect protein, composite probiotics	Fermentation	Functional fermented pet food: The combined use of animal, plant, and insect proteins with corn fiber and composite fermentation reduces lipid exudation during extrusion, lowers biogenic amine formation, enhances antioxidant capacity, improves flavor profile, and supports immunity and anxiety mitigation	China	Functional pet food and preparation method thereof	[68]
White fish meat, glucosamine, chondroitin, methyl sulfonyl-methane, omega-3/omega-6 fatty acids	Mixing, grinding and low-temperature drying	Pet snack: A specific combination and ratio of fish-based protein and joint-supporting functional ingredients, combined with low-temperature, multi-stage drying, improves joint health (including prevention of patellar luxation) while maintaining high palatability and minimizing nutrient degradation	Republic of Korea	Snack composition for improving joint health of companion animals and manufacturing method for snack using the same	[70]
Fish oil, hydrolyzed protein	Centrifuging by decanter	Manufacturing pet food and snacks: Centrifuging the hydrolyzate from which the fish bones are separated into fish oil, hydrolyzed protein, and residual water	Republic of Korea	Separation method of fish and shellfish by-products	[71]
Black soldier fly	Fermentation	Functional pet food: Fermentation of *H. illucens* enhances bioavailability and functionality of insect-derived components, improving digestive efficiency, immune response, and palatability compared with non-fermented insect materials	Republic of Korea	Fermented extract of Hermetia illucens and functional pet food comprising the same	[73]
Sulfur-containing free amino acids, Total free amino acids	Extrusion	Palatability enhancer: A method for preparing cat food with a palatability-enhancing composition	USA	Palatability enhancers comprising amino reactants and carbonyl compounds for use in cat food	[76]
Fish oil, sunflower oil, rapeseed oil, soybean oil, coconut oil, chicken liver hydrolyzate, chicken intestine hydrolyzate, plasma powder, enzyme-hydrolyzed cheese powder, beef seasoning, chicken seasoning, sardine hydrolyzate, shrimp hydrolyzate, tuna hydrolyzate, yeast extract	Drying process by mixing, puffing, baking, or steaming the raw materials	Pet food or pet snacks: The method of manufacturing the prescription-customized functional pet food for companion animals is 100 parts by weight of basic pet food	Republic of Korea	Customized pet food using disease information from genetic testing companies and veterinary hospital examination information and its manufacturing method	[78]
Animal proteins, aquatic proteins, plant-based ingredients	Combining	Cat food: The invention enables individualized nutritional supply by modular pellet design, allowing precise adjustment of nutrient composition according to specific physiological and health needs of cats, while reducing reliance on synthetic additives through the use of natural food-based ingredients	China	Formulation of combined complete cat food	[81]
Meat tissue digest	Hydrolyzed	Palatability enhancer: A method for preparing a meat tissue digest having enhanced palatability to cats	EU	Meat tissue digests having enhanced palatability for use in pet food	[84]
Peptide-based fractions	Optional enzymatic or physicochemical treatment	Health-oriented food products: The disclosed functional material exhibits health-promoting effects when consumed, and its preparation method enables stable incorporation into food-related matrices while retaining functional activity	Japan	Manufacturing method of processed materials and processed products	[85]

**Table 8:** Invention patent-derived evidence on functional pet food innovation: bioactives, processing routes, and claimed health outcomes.

Functional Ingredients	Food Process	Product or Target Application/Major Claim		Country of Patent Applicants	**Patent Name**	**Ref.**
Lactobacillus plantarum JS-1, Lactobacillus paracasei subsp. tolerans JS-2, Schleiferilactobacillus harbinensis JS-3, Lactiplantibacillus pentosus JS-4 (mixed at equal ratio)	Centrifugation, filtration	Functional pet food: Oral administration of the specific probiotic combination alleviates symptoms of atopic dermatitis by reducing epidermal thickening and inflammatory responses, supporting immune modulation and skin barrier recovery		Republic of Korea	Four lactobacillus species with functional activity for improving atopic dermatitis of companion animals, a method of cultivating microbes, its application method in manufacturing functional pet food, and functional pet food as the result	[65]
Desalinated seaweed	Drying and powdering	Cooked pet food composition designed: Inclusion of desalinated seaweed powder provides antioxidant activity, dietary fiber benefits, and nutrient enrichment, while reducing high salt levels to avoid kidney burden, resulting in a functional pet food with health-improving properties		WIPO/Republic of Korea	Cooked pet food composition comprising seaweed and manufacturing method	[66]
Carotenoid (deinoxanthin) extracted, Taurine, Amino acids, Vitamin–mineral premix	Low-temperature hot-air drying	Functional pet food: Low-dose Deinococcus-derived carotenoid (0.01–0.5 wt%) improves antioxidant capacity, protects against brain oxidative damage, and modulates gut microbiota, thereby enhancing vitality and delaying aging in pets		China	Functional pet food based on radio-resistant Deinococcus and preparation method	[67]
*Pediococcus acidilactici* DC-S001	Freeze-drying	Probiotic ingredient: The specific *P. acidilactici* strain is safe (no virulence or antibiotic resistance genes) and effectively increases beneficial bacteria while suppressing harmful taxa, leading to improved gut microbiota structure compared with antibiotics or commercial probiotics		China	A pet probiotic capable of improving canine intestinal flora structure and its application	[69]
Green-lipped mussel, shark cartilage, marigold extract, barley sprout powder, gardenia powder	Low-temperature pasteurization	Liquid functional feed: Liquid stick-type formulation improves palatability and intake rate, reducing selective feeding associated with solid supplements; low-temperature processing preserves functional nutrients while supporting multi-organ health benefits		Republic of Korea	Contains natural extracts of liquid nutritional supplements for pets	[72]
Probiotics, cereal-based protein sources	Extraction, drying, mechanical mixing	Functional pet food: The composition improves digestive efficiency and health indicators without synthetic chemicals, antibiotics, or hormones, while maintaining palatability through fine particle integration		China	Functional pet food for promoting digestion and production process	[74]
Antioxidants, probiotics, isomaltooligosaccharides, digestive enzymes, yucca saponin, β-cyclodextrin	Freeze-drying	Functional pet food: Dual freeze-drying and cyclodextrin inclusion improve the bioavailability and stability of antioxidants and probiotics, enhance palatability, prolong shelf life, and significantly improve vitality and behavioral responsiveness in aged pets compared with commercial supplements		China	Functional pet food for improving activity of old pets and preparation method	[75]
Taurine, lactoferrin, β-glucan	Freeze-drying	Nutritional snack: Provides high taurine content with improved stability, palatability, and long-term shelf life; prevents taurine-deficiency-related disorders such as retinal degeneration, cardiomyopathy, and growth impairment		Republic of Korea	A functional feed composition containing taurine as an ingredient	[77]
Vitamin, mineral, yucca extract, calcium phosphate, methionine, L-lysine	Extrusion and drying process	Extruded product: A health functional pet food characterized by being coated with a natural coating solution composed of a mixture of one or two additives selected from flavoring or sweetener		Republic of Korea	Health functional pet food using deer meat and manufacturing method	[79]
Oil cake	Molding and thermal processing	Functional pet food: Utilization of oil cake improves satiety and nutrient balance, reduces fecal odor and inflammatory responses, and supports immune function while valorizing agricultural by-products		Republic of Korea	Functional pet food composition comprising oil cake	[80]
Microalgae	Fluidized bed drying, spray coating, granulation, tableting	Functional pet food	Stability and processability of fucoxanthin, enabling effective delivery of anti-obesity and anti-diabetic activity	Republic of Korea	Functional feed composition for pets using microalgae, with anti-obesity and anti-diabetes properties	[82]
Agastache rugosa, Haejuksoon, Portulaca oleracea, stevia	Drying	Pet snack: Composition and process claimed to reduce plaque/tartar formation during consumption while maintaining palatability; additional claims of antioxidant/antifungal functionality from botanical additives and potential support for oral health and digestive inflammatory conditions		Republic of Korea	Functional pet food to prevent dental calculus and plaque and method for producing the same	[83]
Fructooligosaccharides	Mixing dough and oven dry	Pet metabolism: Preparation method that produces pet dog biscuits containing FOSs		China	Method for preparing pet dog biscuits with fructooligosaccharides (FOSs)	[86]
Yucca schidigera extract	Controlled heating and mixing	Functional foodstuffs: Reduction in ammonia and sulfur-containing odor compounds in animal excreta by inhibiting urease activity and binding volatile nitrogenous compounds; improves environmental hygiene and animal housing conditions		China	Foodstuffs and methods for their preparation	[87]

## Data Availability

The data presented in this study are available on request from the corresponding author.
